# Feasible H_2_S Sensing in Water with a Printed Amperometric Microsensor

**DOI:** 10.1021/acsestwater.2c00589

**Published:** 2023-04-04

**Authors:** Franc Paré, Rebeca Castro, David Gabriel, Xavier Guimerà, Gemma Gabriel, Mireia Baeza

**Affiliations:** † Department of Chemistry, Faculty of Science, Edifici C-Nord, Universitat Autónoma de Barcelona, Carrer dels Til·lers, 08193 Bellaterra, Spain; ‡ Department of Mining Engineering and Natural Resources, Universitat Politècnica de Catalunya, Avinguda de les Bases de Manresa 61-73, 08240 Manresa, Spain; § Departament of Chemical, Biological and Environmental Engineering, Escola d’Enginyeria, Universitat Autónoma de Barcelona, Carrer de les Sitges, 08193 Bellaterra, Spain; ∥ Instituto de Microelectrónica de Barcelona, IMB-CNM (CSIC), Campus Universitat Autónoma de Barcelona, 08193 Bellaterra, Spain; ⊥ GENOCOV Research Group, Universitat Autónoma de Barcelona, 08193 Bellaterra, Spain; # CIBER de Bioingeniería, Biomateriales y Nanomedicina, Instituto de Salud Carlos III, Madrid, Spain

**Keywords:** amperometric sensor, hydrogen sulfide, inkjet-printed electrodes, single-walled carbon nanotubes, direct ink writing

## Abstract

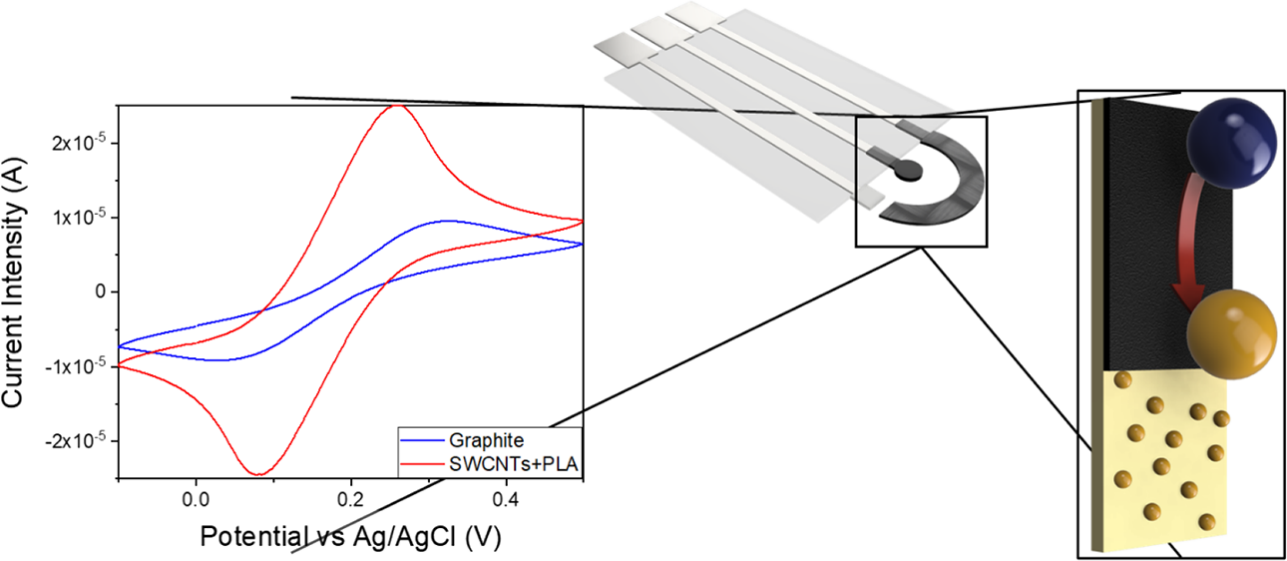

Concern over pollution has led to an increase in wastewater treatment systems, which require constant monitorization. In particular, hydrogen sulfide (H_2_S) is a toxic gas, soluble in water, commonly found in industrial and urban effluents. For proper removal control, fast, durable, and easy-to-handle analytical systems, capable of on-line measurements, such as electrochemical sensors, are required. Moreover, for a proper monitoring of said treatment processes, analysis must be carried out through all steps, thus needing for an economic and highly reproducible method of sensor fabrication. Digital printing have risen in the last few years as technologies capable of mass producing miniaturized electronical devices, allowing for the fabrication of amperometric sensors. Here, a 2 mm^2^ graphite (Gr) electrode, modified with different dispersions of single-walled carbon nanotubes (SWCNTs), poly(vinyl alcohol), poly(diallyl dimethylammonium chloride), and polylactic acid (PLA), is presented as a H_2_S sensor. SWCNTs allow for lower oxidation potentials, higher sensitivity, and a reduced rate of sulfur poisoning, while polymer dispersion of PLA increases mechanical stability and as a result, electrochemical performance. This microsensor presents an optimal pH working range between 7.5 and 11.0, a limit of detection of 4.3 μM, and the capacity to operate on complex matrices for H_2_S contamination detection.

## Abstract 2

In this work, an easy to produce, fast, and reproducible microsensor is presented for the detection and quantification of hydrogen sulfide species in several water environments.

## Introduction

H_2_S is a corrosive and extremely toxic gas, perceptible at very low concentrations as rotten egg smell.^1−3^ H_2_S is naturally emitted from several volcanic and geothermal areas of the world, but it can also be generated in oil and gas production as well as refining industries.^4−6^ Above concentrations of 100 ppm, H_2_S generates saturation of olfactory nerves and poses health risks including respiratory paralysis, irritation, and shock. Above 500 ppm, it has fatal consequences since it affects oxygen uptake in blood.^2^ Furthermore, its exposure is related to Alzheimer’s disease, traumatic brain injury, and hypertension.^7^

While H_2_S frequently appears as gas, it co-exist as H_2_S, HS^–^, and S^2–^ in aqueous media where the percentage fraction of species is related to medium pH (Figure S1).^8,9^ Due to its poisonous nature, environmental hazardous effects, and toxicity for human health, many efforts are focused on development of biotechnological H_2_S removal processes.^10^ However, these treatment processes require adequate tracking systems that can be applied for complex sample measurement.

A variety of methods for H_2_S determination have been established, including chromatography, fluorescence, and optical measurements.^11−14^ However, electrochemical sensors provide a suitable platform due to its rapid detection, high sensitivity, and potential application for on-line measurements.^7,9^ In addition, electrochemical sensors have the advantage of using H_2_S oxidation at low potential for measuring, allowing for a higher selectivity in complex samples.

In recent times, printed electronics have gradually replaced the conventional microtechnology of electrode fabrication.^15^ Among them, direct ink writing (DIW) has generated high interest due to advantages such as low cost, reduced manufacturing time, high reproducibility, mass production, and feasible miniature designs.^16,17^ These features make DIW a suitable alternative for miniaturized electrochemical sensors’ fabrication. Moreover, in contrast to other printing technologies, DIW has a small number of fabrication steps, including substrate and ink preparation, in addition to the printer setup. DIW can be performed under room conditions; thus, a wide range of substrates are compatible for printing, including flexible polymers.^16,18^ In addition, it does not require masks or direct contact between the ink ejector (nozzle, needle, etc.) and the substrate for the printing process, leading to an easy deposition of different materials for multilayer platform fabrication.^17,19^ Moreover, in biotechnological applications, microsensors can be printed on different biocompatible materials and their designs can be adapted to the shape of reactors without substantial modifications.^20^ Therefore, DIW is a promising alternative for H_2_S microsensor fabrication for tracking applications.

Regarding materials used for H_2_S sensor fabrication that can be implemented as inks for any of the DIW processes, one-dimensional carbonaceous nanomaterials such as single-walled carbon nanotubes (SWCNTs) have attracted attention due to high chemical and thermal stability. This material has a large surface ratio and many active sites for H_2_S oxidation.^9,21,22^ In addition, good conductivity, charge transference, and great mechanical strength are SWCNT features that are essential for highly sensitive electrode fabrication.^23^

Furthermore, polymers such as polyvinyl alcohol (PVA), poly(diallyl dimethylammonium chloride) (PDDA), and polylactic acid (PLA) are feasible materials for ink preparation concerning the improvement of its mechanical stability and dispersion. PVA is a polyhydroxy-type polymer, which has hydrophilic, non-toxicity, and biodegradability properties.^23,24^ It can be prepared easily, has a high optical transparency, and has an excellent film-forming capacity.^23,25,26^ On the other hand, PDDA is a water-soluble cationic polyelectrolyte, which can offer anions as charge carriers.^27^ Because of the cyclic quaternary ammonium structure in PDDA, it has an excellent chemical stability and plays an important role in nanoparticle dispersion; thus, it can be used as a combination agent.^24,25,28^ Also, it is an environmentally friendly polymer, which is widely used in industrial applications due to its easy operation. Finally, PLA is a biodegradable polymer, commonly used for 3D printing and biomedical applications, highly insoluble in water, and with good mechanical properties. These properties make it remarkably interesting for monitoring wastewater treatment processes for its resistance to erosion on aqueous samples and its low toxicity toward species present in real samples or complex matrices.

In this work, a H_2_S amperometric microsensor was developed using the cost-effective and reproducible DIW technology to achieve a sensitive, selective, and stable sensor feasible for H_2_S analysis on environmental and bioreactor samples. The optimization of the sensing ink using SWCNTs, PVA, PDDA, and PLA as well as each material’s ratios was studied onto a graphite (Gr) working electrode (WE) to obtain a highly stable and conductive ink, suitable for DIW fabrication processes. Furthermore, the final dispersion selected for ink preparation was used for the H_2_S microsensor fabrication. Even though metal electrodes, such as Au, are a reliable base on which to test modifications, these are unsuitable for the final design as a CE made of metal would still get sulfur poisoning. The H_2_S microsensor was electrochemically characterized, and its pH range of application was evaluated. Finally, its validation was performed using real samples of a sulfate-reducing bioreactor and H_2_S production was measured.

## Materials and Methods

### Reagents and Chemicals

All chemicals were commercially available and were used as received.

For the construction of the Gr integrated electrodes, a screen-printing silver microparticle ink (DuPont 5029 from Dupont, USA) for the conductive tracks, Gr ink (C2030519P4 from Gwent Electronic Materials, UK) for the WE and counter electrode (CE), and a commercial Ag/AgCl ink (DuPont 5874 from Dupont, USA) for the reference electrode (RE) were utilized, and a dielectric ink (LOCTITE EDAG PP 455 BC from Henkel Ibérica, ES) was included for passivation. All inks were printed over a 125 μm-thick polyethylene terephthalate (PET) sheet (Q65HA from DuPont Teijin Films, USA).

For the Gr-WE modification, an ink was prepared from a dispersion of single-walled carbon nanotube carboxylic acid functionalized (SWCNT-COOH), sodium dodecyl sulfate (SDS), PVA, PDDA, and PLA, all purchased from Sigma-Aldrich and anhydrous glycerol (>98%, Honeywell Fluka, USA). For the RE membrane, an ink was prepared by dissolving 10 wt % polyvinyl butyral (PVB) from Sigma-Aldrich, saturated sodium chloride (NaCl, Scharlab, ES) in methanol (Sigma-Aldrich).

Sodium sulfide (98%, Na_2_S·9H_2_O), phosphate buffer saline (PBS) (0.01 M KH_2_PO_4_/Na_2_HPO_4_, 0.0027 M NaCl, 0.137 M KCl, 7.2–7.6 pH), potassium ferricyanide (99%, K_3_(Fe(CN)_6_)), potassium ferrocyanide trihydrate (99%, K_4_(Fe(CN)_6_)·3H_2_O), and potassium chloride (98%, KCl) were all purchased from Sigma-Aldrich. Sodium hydroxide (99%, NaOH) was acquired from Alfa Aesar (ES). For H_2_S stock solution standardization, a Pb^2+^ (0.1 M, standard solution) from Thermo Scientific Orion, USA, was used. All solutions were prepared with deionized water for the Milli-Q system (Millipore, Billerica, MA, USA).

### Instrumentation

A DIW digital material depositor (DMD100 from Kellenn Technologies, FR) was used for the fabrication of bare Gr electrodes.

The printed devices were morphologically characterized by means of a digital microscope (AM4815ZTL from DinoLite, NE) and a field emission-scanning electron microscope (MerlinFE-SEM from Carl Zeiss, GE) with an energy-dispersive X-ray spectroscopy (EDX) analysis system. Electrochemical performance was tested by using a potentiostat μAutolab (PGSTAT204 from Metrohm Autolab BV, NE). Stock solutions of H_2_S were standardized using a commercial S^2–^ ion-selective electrode (Thermo Scientific Orion Star, USA) coupled to a pH/ISE SB90M5 measurement system (SympHony, USA).

Real samples were compared to a SULF-10 commercial H_2_S gas sensor coupled to a X-5 UNIAMP multimeter both from Unisense, DK.

### SWCNTs/Polymer Composite Preparation

Several transductor inks were tested for this sensor fabrication. The first tryout was produced by dispersing SWCNT-COOH (1 wt %) and SDS (0.7 wt %) in deionized Milli-Q water (18.2 MΩ cm). To improve the mechanical stability, polymers were added to the SWCNTs. First, PVA (0.25, 0.5, 0.75, and 1 wt %) was added to the previous composition to test different SWCNTs/polymer ratios. Separately, PDDA (0.5 wt %) was added as PVA previously. Later, PVA (0.4 wt %) and PDDA (0.1 wt %) were mixed and added over the SWCNT dispersion. Finally, a different dispersion with PLA (0.5 wt %) using the previously specified SWCNTs and SDS composition was prepared. Dispersion was achieved by sonicating for 5 min using an ultrasound probe. Between uses, the ink was stored at low temperature (5 °C). Storing the PLA dispersions at low-temperature conditions considerably extends their lifetime. PLA is only soluble in THF, which has a very low boiling point, and is present in only 5 wt % mixed with water and glycerol. If THF evaporates, then PLA starts precipitating before the ink’s usage.

### Electrode Fabrication

To produce each electrode, several materials must be printed over a PET substrate. Since PET films were already pretreated for an enhanced hydrophilicity, no extra treatment was required to achieve good ink adhesion. All printing was carried out in a standard laboratory in ambient conditions. A platform with a three-electrode integrated configuration was designed.

#### Three-Electrode Integrated Platform (Gr-WE, Ag/AgCl-RE, and Gr-CE)

**Figure 1:**
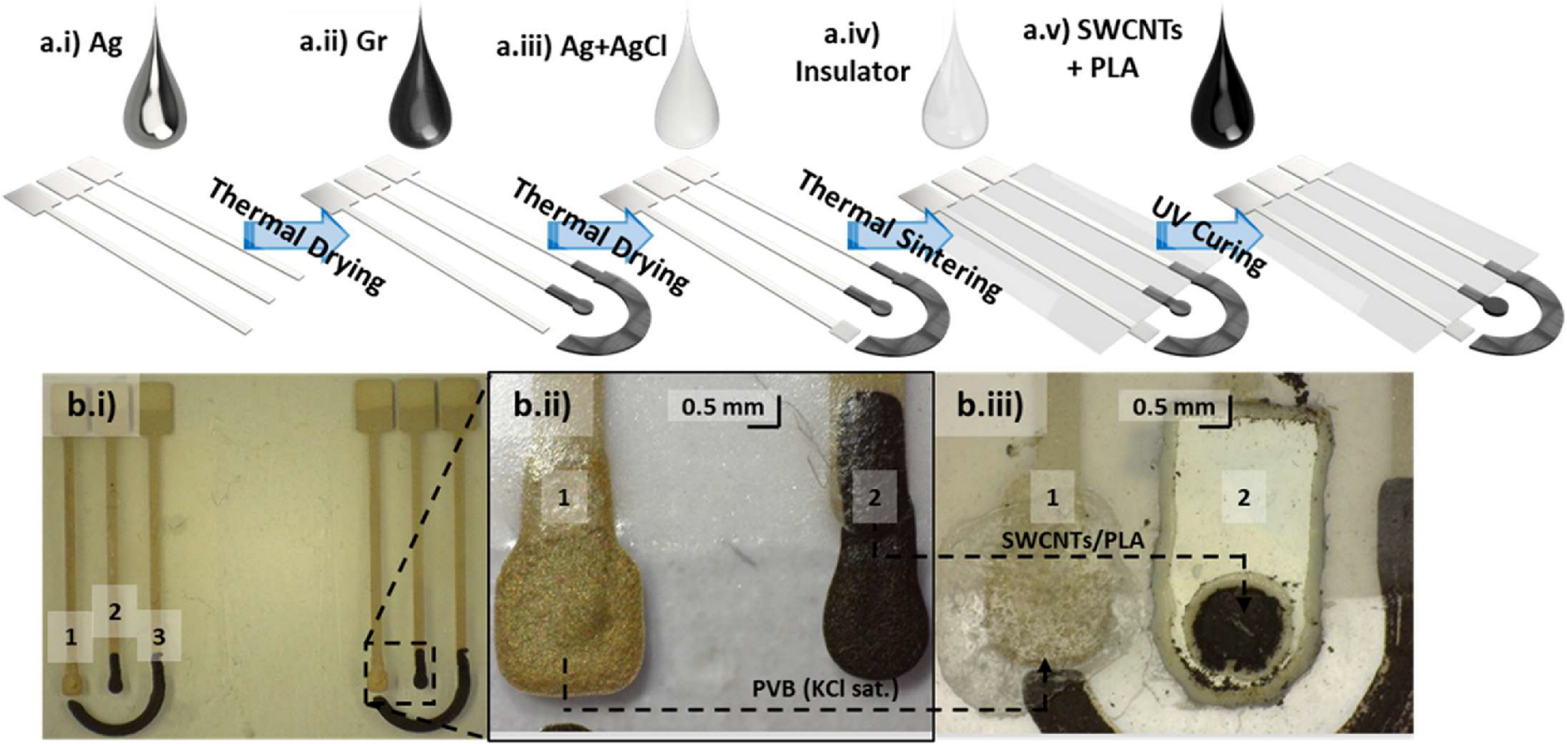
Step-by-step three-electrode integrated configuration (Gr-WE, Ag/AgCl-RE, and Gr-CE) fabrication process by a DMP printer: (a.i) Ag tracks and pads, (a.ii) Gr-WE and Gr-CE, (a.iii) Ag/AgCl-RE, (a.iv) electrode passivation using insulator dielectric, and (a.v) electrode modification by drop casting the SWCNTs/PLA transductor. Also, (b.i) pictures of the final printed platforms 1-Ag/AgCl-RE, 2-Gr-WE, and 3-Gr-CE, (b.ii) close-up of the Ag/AgCl-RE and Gr-WE, and (b.iii) modified electrodes 1-Ag/AgCl-PVB_membrane_-RE and 2-Gr-SWCNTs/PLA-WE.

As shown in Figure 1a.i, the first single Ag layer was deposited using a printing pressure (PP) of 80 kPA and traveling pressure (TP) of 10 kPA at a printing speed (PS) of 100 mm/min to produce the pads and tracks of the WE, RE, and CE. Those inks were then dried at 40 °C for 10 min. Afterward, a layer of Gr ink was deposited to form a 1 mm diameter disc for the WE and the CE (Figure 1a.ii). This was done using a PP of 80 kPa, a TP of 20 kPA, and a PS of 80 mm/min. They were then dried at 40 °C for 10 min. Next, the Ag/AgCl mixture was deposited to form the RE using a PP of 65 kPa, a TP of 10 kPa, and a PS of 80 mm/min (Figure 1a.iii). All inks were then sintered at 150 °C for 1 h or until resistance became lower than 100 Ω.

#### Insulator Printing

A commercial ink was used to cover the tracks with an impermeable dielectric polymer. With it, the silver tracks of the electrodes were protected from corrosion, which could lead to high resistance tracks and faulty measurements. One layer of the LOCTITE dielectric was printed with a PP of 30 kPA, TP of 0 kPa, and PS of 225 mm/min. It was dried by curing under UV light for 30 s (Figure 1a.iv).

#### PVB Membrane Deposition

To grant the electrode better electrochemical stability, the RE was protected with a membrane saturation of Cl^–^. Drops of 1 μL of PVB saturated with NaCl in methanol were casted over the printed RE. This guarantees a constant concentration of Cl^–^ and, as such, potential for the RE as well as protecting it from S^2–^ and other possible chemical species that might attack the electrode.

#### Transducer Deposition

The SWCNT-based inks were drop-casted over the bare Gr electrode area. Drops of 1 μL were used to achieve such small geometries while fully covering the bare electrode beneath. Moreover, transductor inks (SWCNTs/PVA/PDDA and SWCNTs/PLA) were expected to enhance the bare electrodes’ electrochemical capabilities. Finally, the ink was left to dry at room temperature, with no further treatment applied.

### Electrode Characterization

Modification of the Gr electrode was followed by optical microscopy and scanning electron microscopy (SEM). Millimetric images allow for the verification of optimal geometry, possible gaps in the printed surfaces, and long-term variations. SEM was done using a Zeiss Merlin microscope. Samples were prepared by sticking a cut portion of the electrode onto the holder and placing an aluminum tape strip to help in the dissipation of the excess of electrons.

Electrochemical performance was studied by an Autolab potentiostat using cyclic voltammetry (CV) and chronoamperometry (CA) techniques. The external RE of Ag/AgCl (3 M KCl inner filling) and a CE made of a platinum wire, both from ItalSens, PalmSens (NE), were used to complete the system. CV in a 0.01 M [Fe(CN)_6_)]^3–^/[Fe(CN)_6_)]^4–^ solution, which leads to a highly reversible monoelectronic charge transfer reaction, is highly used to study the electrodes’ properties. These include the electrode’s electroactive surface, charge transfer reversibility, and exchange current. Signals were recorded at a scanning speed of 0.01 V/s.

Sensor response was studied using CA, with no stirring while measuring. All calibrations were performed in batch conditions, with subsequent additions of 0.01 M H_2_S standards over 25 mL of PBS adjusted with NaOH to pH 8–8.5.

To standardize H_2_S stock solutions, they were prepared at pH 14, as to form predominantly S^2–^, with a 0.1 M concentration. Then, a potentiometric titration using a standard Pb^2+^ solution and an ion-selective S^2–^ electrode was performed.

Limit of detection (LD) was calculated using the relation
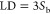
1where *S*_b_ is the standard deviation of the blank.

### H_2_S Response

The electrochemical reaction associated to the measurements is
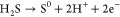
2which has a standard potential^29^*E*^o^ = −0.476 V vs H_2_/H^+^ ≈ −0.080 V vs Ag/AgCl. Consequently, the reaction is spontaneous under standard conditions and stock solutions must be prepared daily.

The concentration at the sensor’s surface and intensity current can be related through Cottrell’s equation:
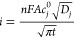
3with *c_j_*^0^ as the species activity at the electrode’s surface, if performed under diffusion conditions. *F* is Faraday’s constant, *A* is the electrode’s electroactive area, *n* is the number of electrons exchanged, *D_j_* is the diffusion coefficient of the reacting species, and *t* is time. Measured at a given time, *i* vs *c* results in a linear correlation.

The parameters for the measurements are the response time (*t*_r_), which is the time required to accurately carry out measurements at 20 s. Also, given that random noise is always present in measurements, the best option is to average several signals. Since the signal is time-dependent, the averaged values must be taken in a short range of time to avoid reflecting this dependence. As such, a 0.1 s gap (10 values) is used for the chronoamperometric measurements. Finally, 0.1 V vs Ag/AgCl was used as the working potential for the H_2_S measurements (Figure S2).

### Interference Study

To measure the possible interference of other species commonly found in water samples, different solutions were spiked with stocks of known concentration. All solutions were measured with the same sensor consecutively and by triplicate.

### Sample Preparation

Samples, both spiked and real, were prepared simultaneously to ensure minimum variability. Each was adjusted to the optimal pH for the commercial or our sensor, respectively. Spiked samples, consisting of Milli-Q and tap water, were doped with H_2_S to known concentrations. Real samples come from a sulfate-reducing reactor and are expected to contain a lower concentration of hydrogen sulfide at the entrance and a higher one at the exit.

## Results and Discussion

### Optimization of Composite Composition

Different dispersions of SWCNTs on polymers (SWCNTs/PVA, SWCNTs/PDDA, SWCNTs/PVA/PDDA, and SWCNTs/PLA) have been studied within this work to overcome the mechanical limitations that the SWCNTs present. SWCNTs are hard to disperse in aqueous solvents due to their low polarizability and require a stabilizing agent. In this case, SDS was used as a stabilizing agent to increase the dispersion quality.^30^ However, the problem was rather with the stability once the ink had dried and the SWCNTs had been deposited over a different material. Thus, the polymers were added as a support to the SWCNTs. However, as to not decrease the electroanalytical response, the ratio of the polymer could not exceed that of the SWCNTs. Therefore, the chosen SWCNTs/polymer ratios tested were 1/0.25, 1/0.5, 1/0.75, and 1/1. To determine the most optimal one, several dispersions were drop-casted over a PET substrate and they were observed after 72 h submerged in water to test their improvement of printability (Figure S3) compared to the SWCNTs alone. After 72 h, a pure SWCNT deposition is completely removed; thus, this was the minimum time to overcome the mechanical stability.

From the durability tests, it was decided that a SWCNTs/polymer ratio of 1/0.5 would work the best as higher polymer ratios lead to very few SWCNTs being deposited, and lower ones did not improve the stability enough.

On one hand, PVA composites with SWCNTs were discarded as the rapid apparition of large aggregates in the prepared inks was observed. These aggregates made the composites easily peel off (Figure S3) and not last enough for a full electrochemical characterization. On the other hand, PDDA composites present their own oxidation and reduction signals (Figure S4). The oxidation peak potential around 0.08 V vs Ag/AgCl has the direct inconvenient that interferes with the analyte measurements as H_2_S is oxidized at −0.08 V vs Ag/AgCl and gradually degraded the sensor. However, the degree of dispersibility and stability of dispersions of the SWCNTs in PDDA was adequate. Thus, it was decided that mixing at the same time could be interesting to improve the ink’s properties. The SWCNTs/PVA:PDDA ratio was maintained in 1/0.5 (total SWCNTs/polymer), and from all the tested dispersions, 1/0.4/0.1 was the only ratio that both minimized the formation of aggregates and simultaneously prevented the apparition of significant currents from the oxidation of PDDA.

As the initial problems with aggregates were not fully solved, PVA and PPDA were substituted for PLA. The first test with PLA, over a PET substrate using a 1/0.5 SWCNTs/PLA ratio, yielded far better results than any previous composite in its durability when submerged in water for 72 h (Figure S5).

### Electrochemical Performance Evaluation

**Figure 2:**
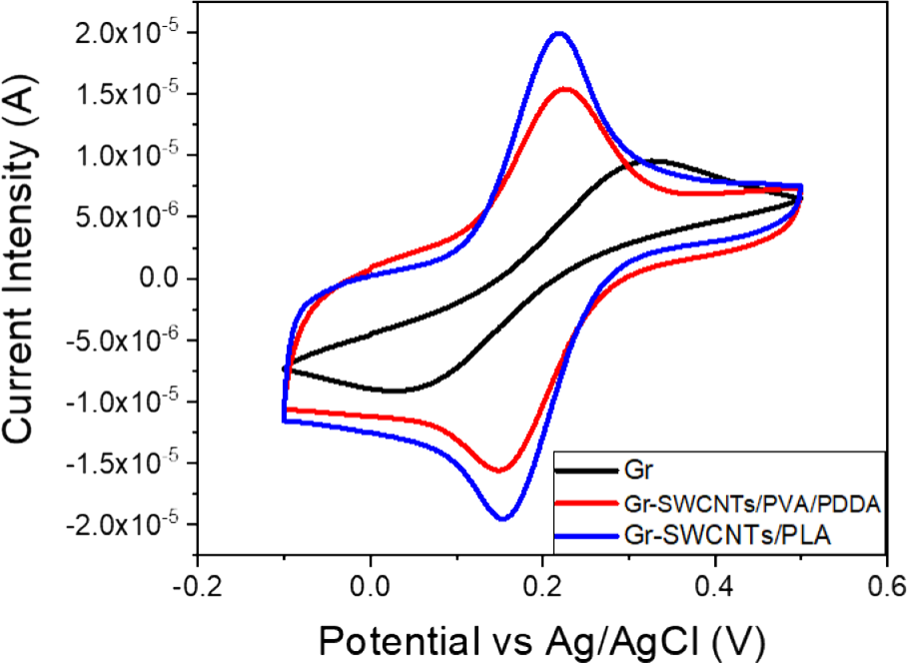
Electrochemical response of the printed bare Gr electrode and modified with SWCNTs/PVA/PDDA or SWCNTs/PLA in a solution of 0.01 M [Fe(CN)_6_)]^3–^/[Fe(CN)_6_)]^4–^ at a scanning speed of 0.01 V/s. Intensity currents are normalized with the geometrical area.

We assess the electrochemical response improvement by modifying the bare Gr electrode with the previously tested ratios of 1/0.4/0.1 SWCNTs/PVA/PDDA and 1/0.5 SWCNTs/PLA composites. The peak current, voltage separation, and the calculated electroactive area, calculated using Randles–Ševčík (Table S1) of the performed voltammograms (Figure 2), were used as the parameters of reference for comparison.

As expected, the addition of SWCNTs over the bare electrode (Gr) increases the electroactive area (Table S1), shown as an increase in oxidation and reduction current intensities. This is clearly seen with the modification of Gr with SWCNTs/PLA. In that case, the electroactive to geometric area is almost 2 times bigger with respect to bare Gr electrodes. On the other hand, for Gr- SWCNTs/PVA/PDDA, the ratio is approximately the same, though still larger than that of Gr. Potential peak differences of 75 and 59 mV were obtained when Gr-SWCNTs/PVA/PDDA and Gr-SWCNTs/PLA electrodes were used, respectively, indicating that electron transfer reactions presented a greater degree of electrochemical reversibility than for the Gr electrode (239 mV). Thus, the smallest peak separation is achieved when SWCNTs/PLA are drop-casted over Gr. This electrode presents the best electrochemical behavior and the most reversible (59 mV) electron-transference process.

### Electrode’s Structure and Morphology

**Figure 3:**
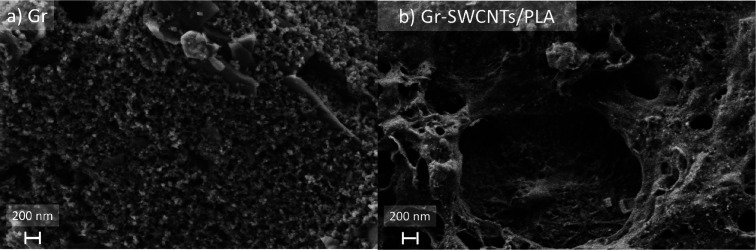
Morphological characterization by SEM of the (a) Gr electrode and (b) Gr-SWCNTs/PLA electrode’s surface.

SEM micrographs of the bare electrode and the final chosen material, as shown in Figure 3, allow us to observe the change in morphology between Gr (a) and Gr-SWCNTs/PLA (b). As can be seen, Gr is much flatter, even though it still presents many small moieties, than Gr-SWCNTs/PLA. The latter one has a much rougher surface, with pores and crevices. The polymer helps in the formation of irregular structures, and the images reveal a random distribution of entangled SWCNTs perfectly integrated in the polymer matrix improving the SWCNTs’ own rugosity. This explains the electrodes’ larger electroactive surface compared to the bare electrodes. The SWCNTs/PVA/PDDA composite, upon deposition over the electrode, results in the formation of an external layer of the polymer, covering the outmost CNTs. As a non-conductive material, it was not possible to obtain any quality image of the surface at a nanoscale. The best results were obtained by applying a very high voltage, with which the electrons managed to sufficiently penetrate the external membrane and get very little detailed images of the inner surface (Figure S6A). Nevertheless, little information can be obtained from these micrographs. The only alternative was metallizing the electrodes. Still, a layer at least 20 nm thick was needed to obtain quality images (Figure S6B).

Confocal microscopy measures the roughness of a surface. In our case, this is correlated to the electroactive area. As this was an important criterion upon choosing the material, further confirmation of the difference between SWCNTs/PVA/PDDA and SWCNTs/PLA helps in explaining this result. Profiles of the surfaces are obtained for each electrode and, from there, the value of roughness calculated (Figure S7). These profiles show that the roughness of SWCNTs/PLA is the highest among all electrodes, with Gr being the smoothest. This is the same tendency that was seen previously with electroactive areas using CVs (Table S1). Thus, the correlation is as predicted.

Infrared spectroscopy can be used to differentiate the presence of the polymers added in each mixture. This is purely a qualitative measurement; nonetheless, it helps in highlighting the differences between the polymers PVA/PDDA and PLA. As can be seen in Figure S8, SWCNTs present an overall higher transmittance compared to the polymer mixtures. The PVA/PDDA composite presents a very broad band between 3000 and 3600 cm^–1^, which is characteristic of the O–H and N–H bonds present in PVA and PDDA, respectively. In contrast, for the composite with PLA, this band is barely visible, as is in the case of pure SWCNTs. Given that PLA does not have the O–H or N–H bonds, a smaller signal is expected. The small band that appears belongs to the surfactant used in the dispersion, SDS, which also presents an O–H bond after dissolving due to the substitution of the sodium cation for a proton. The lower transmittance for the composites suggests thicker layers than pure SWCNTs, despite the same volume being used.

### SWCNTs/PVA/PDDA and SWCNTs/PLA Analytical Response

First, to determine which potential H_2_S should be measured, linear sweep voltammetry (LSV) was performed at 5 mV/s, from 0 to 0.5 V vs Ag/AgCl, in a 10 mM (pH 8.5) H_2_S solution (0.1 M KCl), and the represented voltammogram is obtained using a SWCNTs-PLA sensor (Figure S2). This is to find an applied voltage sufficiently high to allow the electrochemical H_2_S oxidation to take place but low enough to offer a high selectivity toward other ions. The maximum current is achieved around 0.17 V vs Ag/AgCl before the reaction is limited by mass transfer. However, at around 0.1 V vs Ag/AgCl, the current is at half height of the maximum. As selectivity is achieved by using the smallest oxidation potential possible, this was found as a compromise value between selectivity and sensitivity. There is enough signal to detect H_2_S at the desired concentrations, with a reduced sulfur-poisoning rate and an increased selectivity.

**Figure 4:**
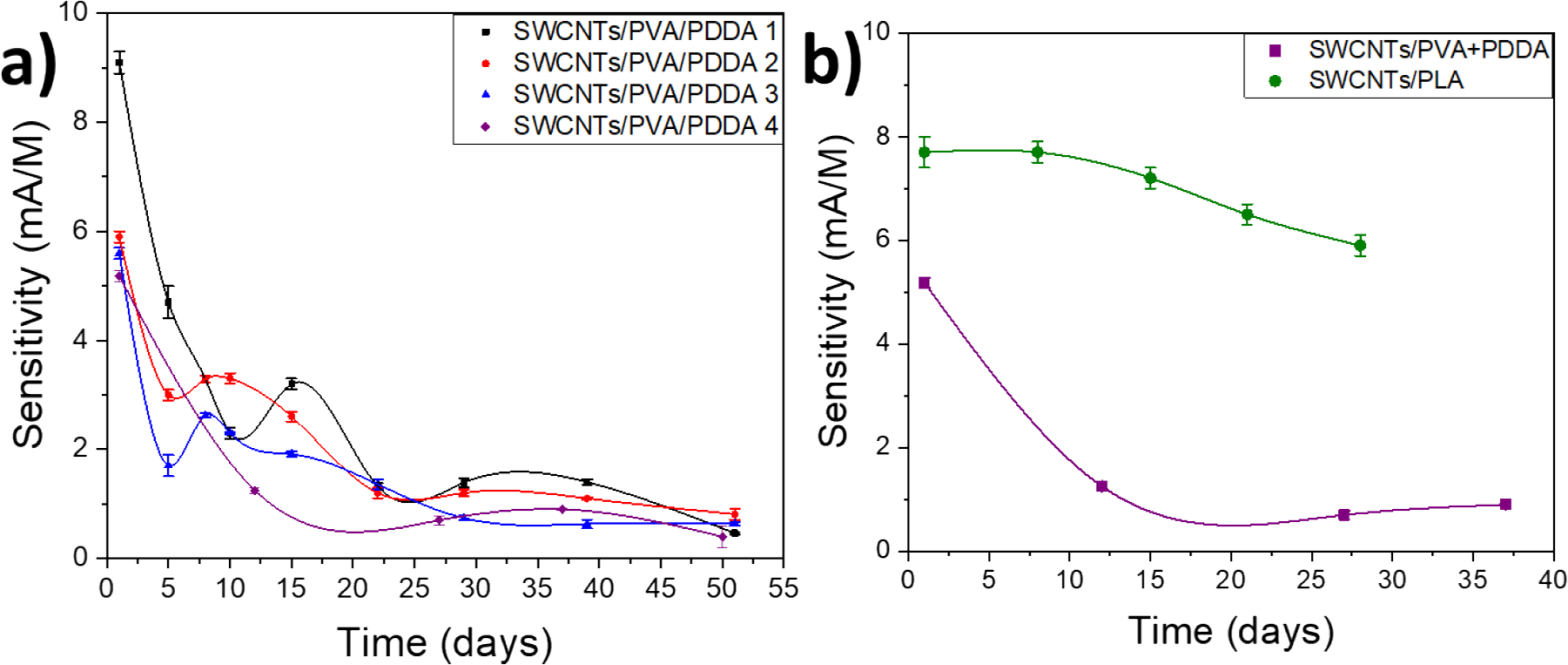
(a) Gr-SWCNTs/PVA/PDDA sensor stability and its decay along the first 20 days of usage. (b) Sensitivity comparison evolution of two Gr electrodes, one modified with SWCNTs/PVA/PDDA and another with SWCNTs/PLA.

Following the chronoamperometry method with the parameters previously described, several calibrations of four different Gr-SWCNTs/PVA/PDDA sensors were performed. As calibrations were repeated thrice (*n* = 3), after several days, it was found that the SWCNTs/PVA/PDDA dispersion lacks any long-term stability (Figure 4a). After days of measurements, under the previously described conditions, sensors lose up to 80% sensitivity (from ∼6 to ∼1 mA/M), with shorter linear ranges and higher limits of detection as calibrations were done. As PVA and PDDA would progressively erode from the electrode, a large portion of the SWCNTs was also lost. This caused the decay in response toward H_2_S. Consequently, PLA was proposed to overcome the solubility problems observed with PVA/PDDA. To keep the ratio that was previously found as optimal, a dispersion of 1/0.5 SWCNTs/PLA was chosen for the final validation for H_2_S sensing. As can be seen in Figure 4b, the sensitivity of the new SWCNTs/PLA dispersion decreases much slower, from a sensitivity of 7.7–5.9 mA L mol^–1^ (23%), than in the SWCNTs/PVA/PDDA one. Given the stability results and the better ratio of the electroactive area, PLA is chosen to carry on with the final sensor.

### Three-Electrode Integration

To complete the full integration of the Gr electrode as the WE, a functional RE and CE were integrated. The RE is made of a Ag/AgCl paste and protected from sulfur attacks with a PVB membrane^31^ that is NaCl-saturated to increase the stability of the electrode by keeping a constant concentration of Cl^–^. To verify that the integrated CE and RE were good enough to replace the previously used commercial ones, CVs were performed under the same conditions (Figure S9) using a Gr. Results showed no variations in the intensity, even as the CE is swapped, but a decrease in peak separation when using the integrated RE compared to an external commercial reference electrode. As such, the systems are not only interchangeable perfectly, but the integrated electrodes also lower the overpotential, improving the electrochemical performance sensor.

### Optimizing the Working pH

**Figure 5:**
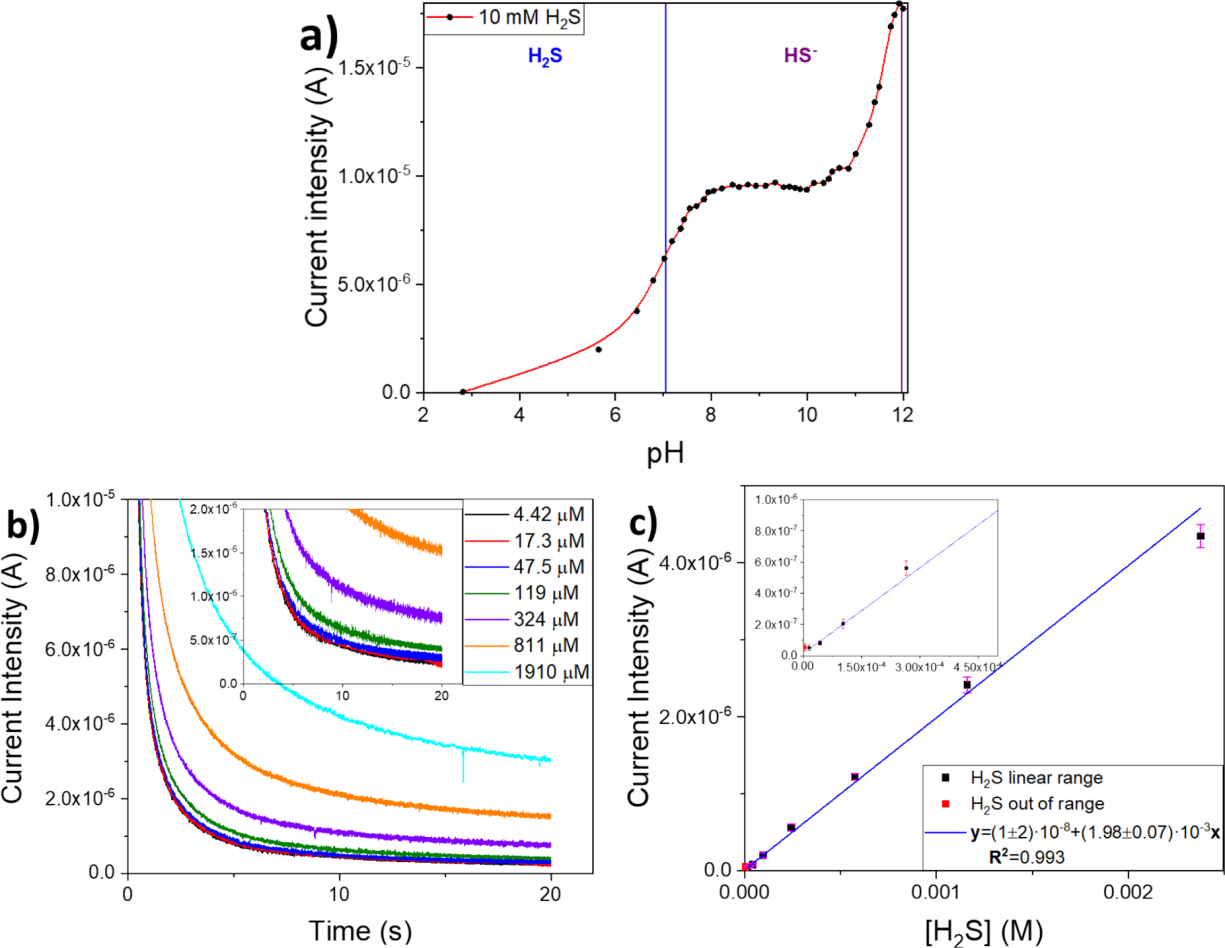
(a) Characterization of the response of the Gr-SWCNTs/PLA integrated sensor to H_2_S species on a 3–12 pH range. (b) Chronoamperometry measurements at different H_2_S concentrations with a close-up into the smaller ones (inset). (c) Calibration curve (*n* = 3) of the Gr-SWCNTs/PLA-modified sensors for H_2_S oxidation at an 8 < pH < 9 range with a zoom-in into the smaller concentrations (inset).

Of even greater importance was the response variance at different pH, given that H_2_S partially appears as a gas at acidic pH (Figure S1). Under this condition, stripping can occur. As a result, all measurements must be carried out in a limited amount of time. Thus, finding an optimal range of operation is of paramount importance. With the optimized conditions, oxidation current is measured at different pH values at a fixed analyte activity and equal time (Figure 5a). As can be seen, the response presents the smallest variations in a pH range between 7.5 and 11.0, which corresponds to a dominant presence of HS^–^ over H_2_S and S^2–^ (Figure S1). In consequence, the sensor will operate at optimal sensitivity in the pH-independent region. Nonetheless, it would still be capable of measuring at pHs lower than 7.5, so far as the pH dependence is quantified and sensor response compensated accordingly by normalizing.

### Electroanalytical Performance

With all parameters considered, it was proceeded to evaluate the response of the electrode to variations of the H_2_S concentration. Calibrations were carried out at a starting pH of 8–8.5, using a PBS buffer, and NaOH to adjust pH, to ensure that there was no significant change in the response of the sensor. As previously stated, stock solutions need to be prepared every few hours to prevent a severe decay of the starting concentration. However, by preparing stock solutions at a pH of 12, oxidation is much slower, allowing them to last unaltered through the entire calibration procedure.

Different concentrations, prepared on the spot by additions of different volumes of stock solution, were measured in separated chronoamperometries with no stirring (Figure 5b). The signal was taken between 19.9 and 20 s in a range from 0 to 2380 μM H_2_S. Each measurement was repeated three times (*n* = 3). The sensor shows a good linear correlation between concentrations of 16.3 and 2380 μM H_2_S and a LD of 4.3 μM (Figure 5c).

**Figure 6:**
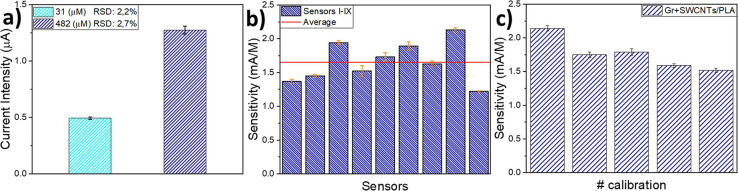
Sensor’s performance study. (a) Repeatability: repeated measurements (*n* = 5) of the same concentration, using the same electrode, consecutively. (b) Reproducibility: comparison of the sensitivities of the first calibration of nine different sensors and their average, built in different days but under the same conditions. (c) Short-term stability: decrease in sensitivity when several calibrations (*n* = 5) were performed in a single day (120 measurements). Experimental errors were calculated as standard deviation.

Additionally, repeatability, reproducibility, and short-term stability were also studied to fully characterize the Gr-SWCNTs/PLA integrated sensor response (Table S2). Repeatability consists of the consecutive measurement of different samples of equal concentration. After five measurements at 31 μM, with an RSD of 2.3%, and at 430 μM, with an RSD of 2.7%, it was proven that, for a given sensor at a certain concentration, there was no significant signal changes (Figure 6a). Moreover, this proves that, while a working potential of 0.1 V vs Ag/AgCl could lead to noise due to variations in the applied voltage, this noise is small and does not affect the measure.

Data shows that the optimized method of measurement allows for a small dispersion of signal values when measuring a specific analyte’s concentration. Reproducibility is the calibration of several electrodes, equally fabricated and measured, under the same conditions, consecutively. With an average sensitivity of 1.65 mA/M and a standard deviation of 0.29 mA/M (18%), the fabrication method of the electrodes bears some difference between their responses (Figure 6b). This is most likely attributable to the manual nature of the last fabrication step by drop casting the SWCNTs/PLA dispersion. Short-term stability consists of continuous calibrations (analyzing *n* = 9 H_2_S concentrations) of a sensor for 1 day. As can be seen in Figure 6c, sensitivity is only lightly affected by consecutive measurements, going from 2.14 to 1.52 mA/M or decreasing its response by 29% over 120 measurements. This shows that sulfur poisoning is close to negligible, and the sensor suffers little strain over continuous usage.

### Interference Study

To assess the selectivity of the sensor, several species commonly present in water samples were measured (Figure S10). All the measured species give a signal of the same magnitude compared to that of the LD of H_2_S. When measured at an optimal pH and equal concentration, none of the interferents gives a current close to that of the analyte. This corroborates that by using a low working potential, the sensor manages to achieve good selectivity for the target medium.

### Real Sample Study

The final assessment of the applicability of the sensor was studied by measurements on spiked and real samples of increasing matrix complexity. To contrast the results, they are compared with a commercial H_2_S sensor.

Sensors are calibrated right before measuring the samples. Results show (Table 1) that the sensor is capable of quantitatively measuring H_2_S in complex matrix samples since all the calculated *t*-Student values are lower than the corresponding limit for 2 degrees of freedom at a confidence interval of 95% (2.92).

**Table 1:** Comparison Response between Our Printed Sensor and the Commercial H_2_S Sensor^a^

	sample type	commercial sensor [H_2_S] (μM)	measured [H_2_S] (μM)	*t*-test (*n* = 2, 95%)
printed sensor	Milli-Q water	180.6 ± 0.3	193.6 ± 0.1	0.62
	tap water	197.1 ± 0.6	210.4 ± 0.7	1.89
	reactor media	178.5 ± 0.7	206.9 ± 0.5	2.82

a The measurement error was expressed as the standard deviation (*n* = 2).

## Conclusions

In this work, a miniaturized, fully printed H_2_S sensor is presented. The fabrication and characterization steps are thoroughly described. Results show that printing technologies can be used to produce electroanalytical devices. In particular, an easy and cheap to mass-produce sensor capable of operating for over 25 days with minimal sensitivity decrease is shown. Nevertheless, future work can further improve carbon-based material inks for fully printable electrodes. Still, the modifications applied using a SWCNTs/PLA ink over bare Gr electrodes show a clear improvement in sensitivity (up to 2.2 mA/M in the best sensors) and sulfur-poisoning resistance, performing up to 135 measurements with less than 30% signal loss. PLA helps to increase the mechanical resistance of the Gr-SWCNT sensor, resisting erosion far longer than 72 h of the SWCNTs by themselves. This allows the sensor to continuously operate for a few days or during measurement-intensive periods without needing replacement. Moreover, the optimal pH range for measurements, the integration of all the electrodes into a single platform, and the high selectivity achieved by the low oxidation potential of H_2_S make it capable of working under real conditions with complex samples matrices.

## Supporting Information Available

Hydrogen sulfide species in aqueous media (Figure S1); study of the electroactive area and voltage separation (Table S1); determination of working potential to oxidize H_2_S using a SWCNTs-PLA sensor (Figure S2); images of different proportions of SWCNTs/PVA (Figure S3); CV of a Gr electrode modified with SWCNTs/PDDA 1/0.5 (Figure S4); photographs of the deposition of SWCNTs/PLA (Figure S5); micrographs obtained by SEM of PVA/PDDA electrodes (Figure S6); surface 3D profiles obtained using confocal microscopy to study the roughness (Figure S7); infrared analysis for qualitative differentiation of the composition (Figure S8); electrochemical characterization and comparison of the integrated electrode (Figure S9); interference study of several species common in water samples (Figure S10); study of repeatability, reproducibility, and short-term stability (Table S2) (PDF)
