# Supplementary material for: Feasible H2S Sensing in Water with a Printed Amperometric Microsensor
Source: ACS ES T Water. 2023 Apr 4;3(4):1116–25. doi: 10.1021/acsestwater.2c00589 (PMC10753653; doi:10.1021/acsestwater.2c00589)
Supplement: Supplementary file 1 — ew2c00589_si_001.pdf [file ew2c00589_si_001.pdf]

Feasible H_2S sensing in water with a printed amperometric microsensor

Franc Paré[1,5], Rebeca Castro[2], David Gabriel[3,5], Xavier Guimerà[2], Gemma Gabriel[4,6], Mireia Baeza[1,5,*]

[1] *Department of Chemistry, Faculty of Science, Edifici C-Nord, Universitat Autònoma de Barcelona, Carrer dels Til·lers, 08193 Bellaterra, Spain*

[2] *Department of Mining Engineering and Natural Resources, Universitat Politècnica de Catalunya, Avinguda de les Bases de Manresa 61-73, 08240 Manresa, Spain*

[3] *Departament of Chemical, Biological and Environmental Engineering, Escola d'Enginyeria, Universitat Autònoma de Barcelona, Carrer de les Sitges, 08193 Bellaterra, Spain*

[4] *Instituto de Microelectrónica de Barcelona, IMB-CNM (CSIC), Campus Universitat Autònoma de Barcelona, 08193 Bellaterra, Spain*

[5] *GENOCOV Research Group, Universitat Autònoma de Barcelona, 08193 Bellaterra, Spain*

[6] *CIBER de Bioingeniería, Biomateriales y Nanomedicina, Instituto de Salud Carlos III*

* Correspondence: mariadelmar.baeza@uab.cat

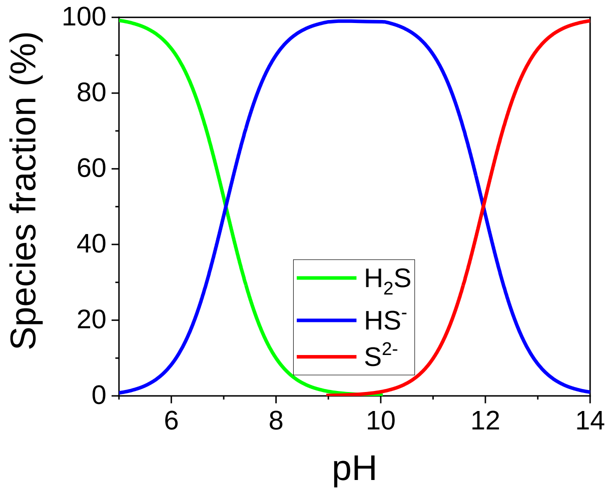

Figure S1. Species proportion as a factor of the media pH. The ionic species, HS^- and S^{2-} predominate at basic pHs.

Table S1. Study of the electroactive area and voltage separation.

Material	Intensity peak (μA)	Geometric Area (mm^2)	Electroactive Area (mm^2)	ΔE (mV)
Gr	7.12	1.005	3.140	239
Gr-SWCNTs/PVA/PDDA	11.76	1.487	5.153	75
Gr-SWCNTs/PLA	16.45	1.230	7.207	59

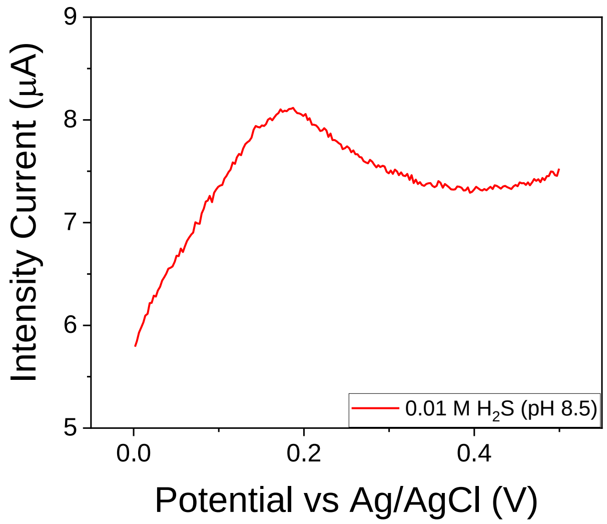

Figure S2. LSV for the determination of working potential to oxidize H_2S using a SWCNTs-PLA sensor.

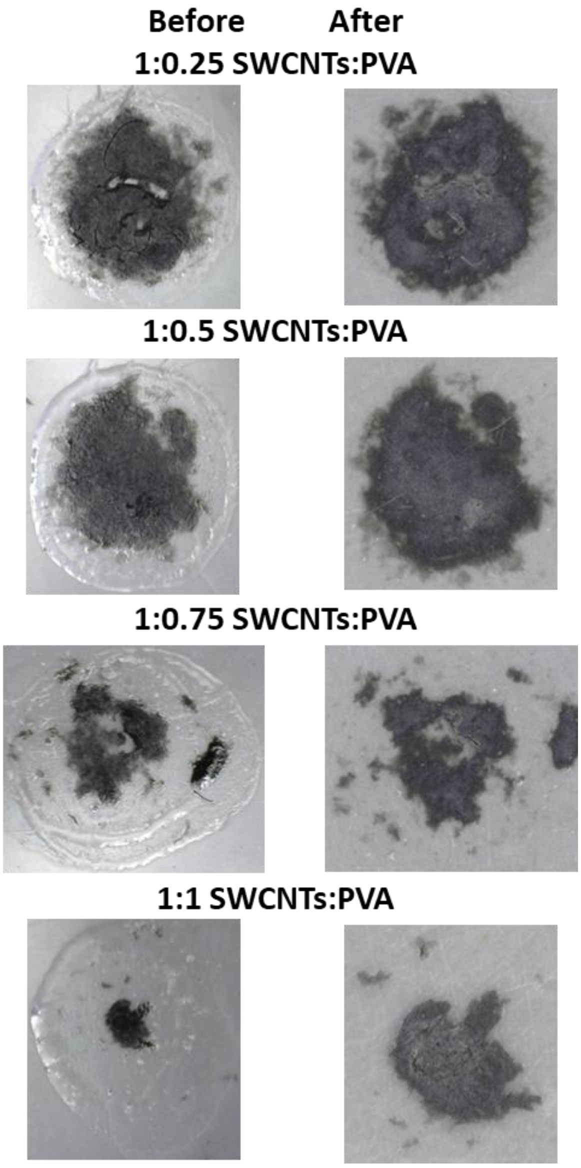

Figure S3. Photographs of the deposition of different proportions of SWCNTs/PVA and their morphology after being submerged in water for 72 h to determine the most optimal composition.

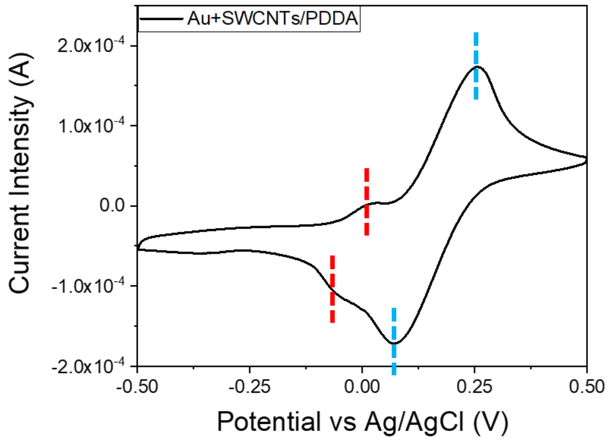

Figure S4. CV of a Gr electrode modified with SWCNTs/PDDA 1/0.5. As can be seen (red dash) PDDA oxidates at very small potentials, blue dashes belong to the reversible oxidation/reduction of 0.01 M $[Fe(CN)_6]^{4-}$/ $[Fe(CN)_6]^{3-}$solution at scan rate 0.01 V/s.

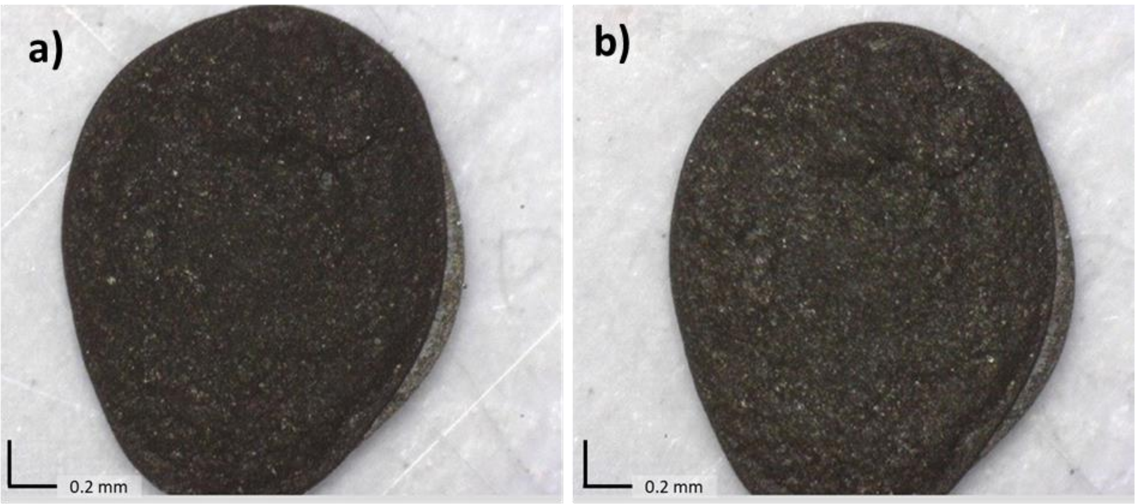

Figure S5. Photographs of the deposition of SWCNTs/PLA and their morphology before (a) and after (b) being submerged in water for 72 h.

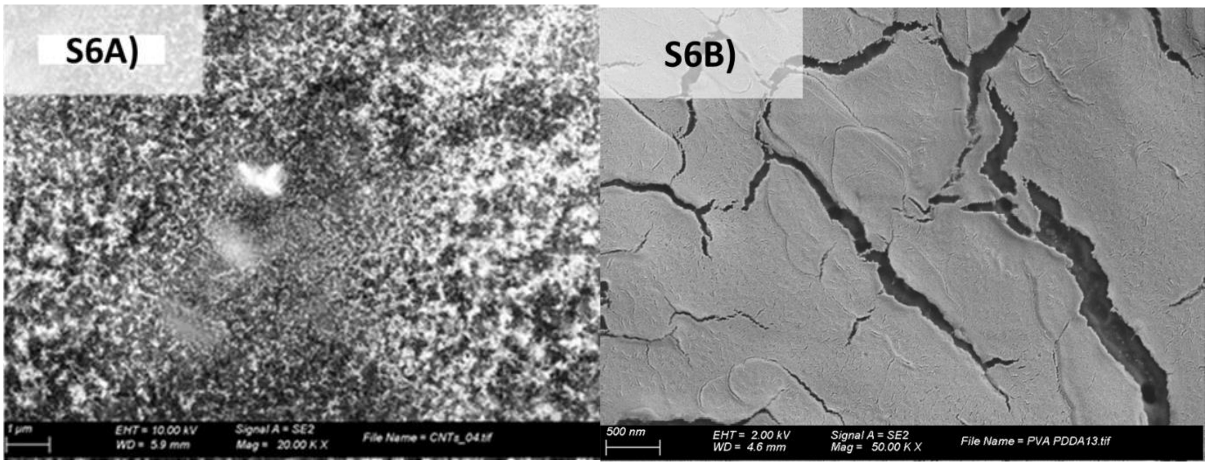

Figure S6. Micrographs obtained by Scanning Electron Microscopy of a A) SWCNTs/PVA/PDDA electrode and B) the metalized SWCNTs/PVA/PDDA.

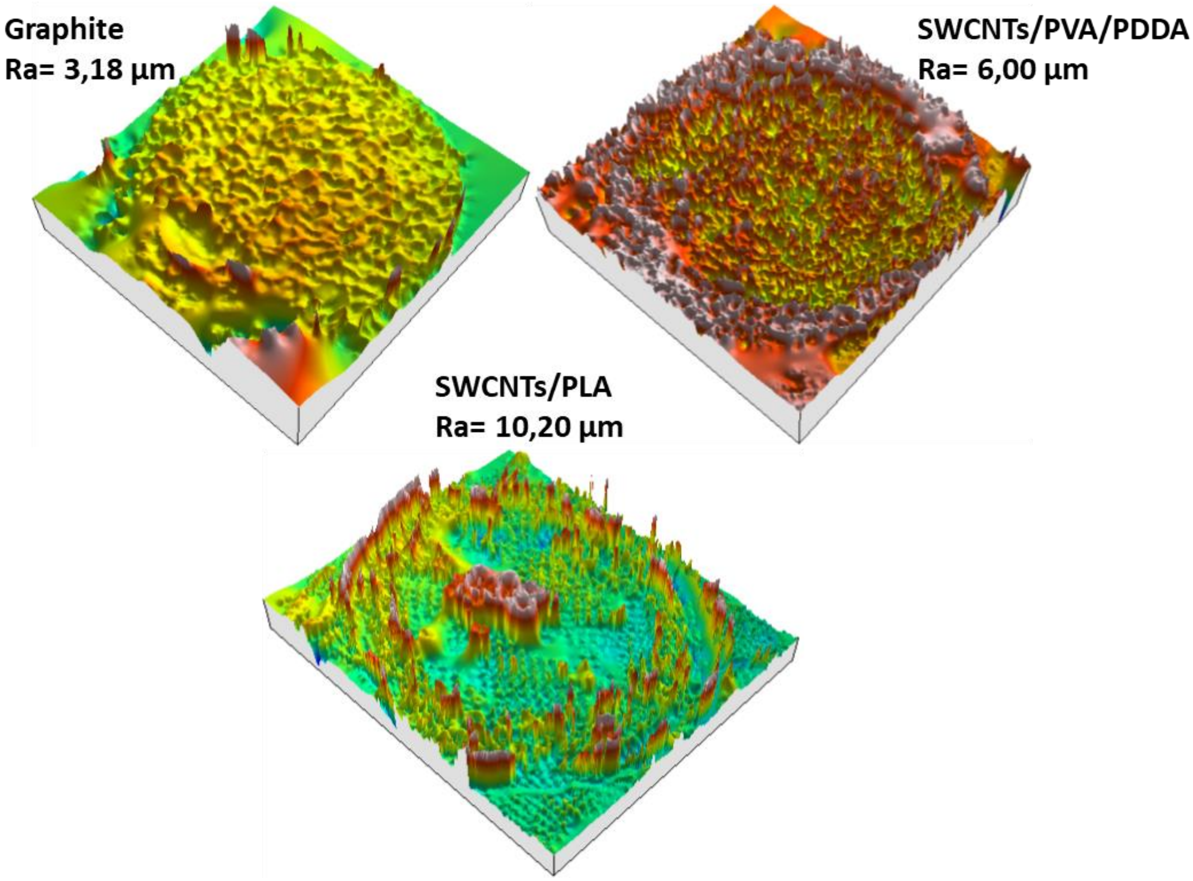

Figure S7. Surface 3D profiles obtained using a confocal microscopy to study the roughness of A) Graphite, B) SWCNTs/PVA/PDDA and C) SWCNTs/PLA.

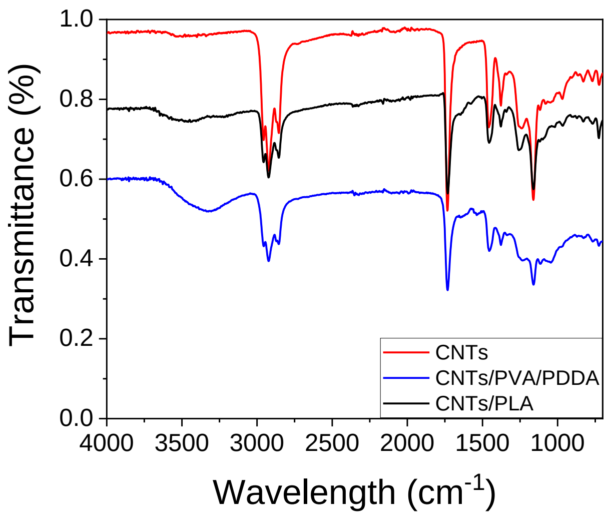

Figure S8. Infra-red analysis for qualitative differentiation of the composition of A) SWCNTs, B) SWCNTs/PVA/PDDA and C) SWCNTs/PLA.

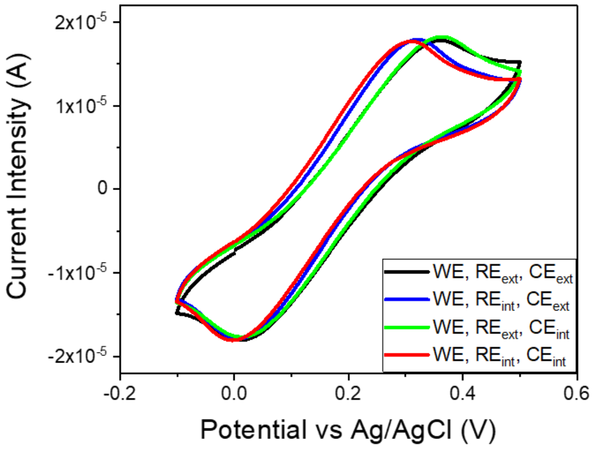

Figure S9. Electrochemical characterization and comparison of the integrated electrodes using a Gr under a 0.01 M $[Fe(CN)_6]^{4-}/[Fe(CN)_6]^{3-}$ solution at a scan rate of 0.01 V/s.

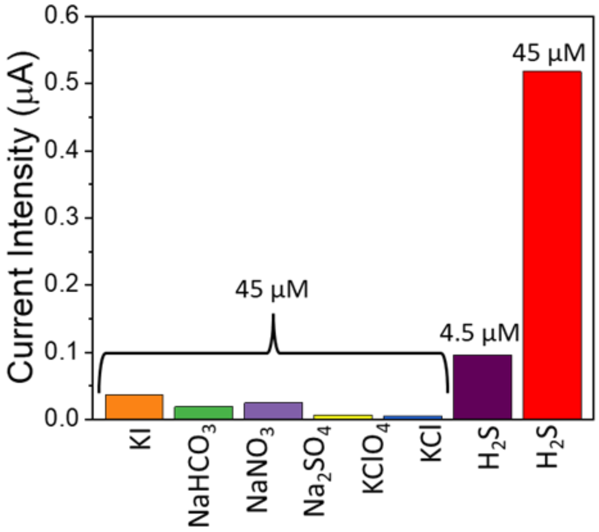

Figure S10. Interference study of several species common in water samples.

Table S2. Study of repeatability, reproducibility, and short-term stability. The sensitivity error was expressed as the standard deviation (N=3).

Study	Measurement	[H_2S] (μM)	Signal (μA)	Sensitivity (mA/M)	RSD (%)
Repeatability	1	31	0.483		2.30
	2		0.498		
	3		0.510		
	4		0.490		
	5		0.484		
	1	430	1.23		2.74
	2		1.26		
	3		1.31		
	4		1.30		
	5		1.28		
Reproducibility	1	16.3-1830		1.37±0.03	17.9
	2			1.45±0.02	
	3			1.94±0.03	
	4			1.52±0.08	
	5			1.73±0.06	
	6			1.89±0.06	
	7			1.63±0.04	
	8			2.13±0.03	
	9			1.22±0.01	
Short-term stability	1	16.3-1830		2.3±0.1	12.0
	2			1.94±0.07	
	3			1.90±0.03	
	4			1.80±0.06	
	5			1.69±0.05	
